# Pancreatic Lipase Inhibition by *C*-Glycosidic Flavones Isolated from *Eremochloa ophiuroides*

**DOI:** 10.3390/molecules15118251

**Published:** 2010-11-16

**Authors:** Eun Mi Lee, Seung Sik Lee, Byung Yeoup Chung, Jae-Young Cho, In Chul Lee, So Ra Ahn, Soo Jeung Jang, Tae Hoon Kim

**Affiliations:** 1 Advanced Radiation Technology Institute (ARTI), Korea Atomic Energy Institute (KAERI), Jeongeup 580-185, Korea; E-Mails: leeeunmi@kaeri.re.kr (E.M.L.); sslee@kaeri.re.kr (S.S.L); bychung@kaeri.re.kr (B.Y.C.); 2 Department of Applied Life Sciences, Chonbuk National University, Jeonju 561-756, Korea; E-Mails: soilcosmos@jbnu.ac.kr (J.Y.C.); 3 Senior Industry Cluster Agency, Youngdong University, Youngdong 370-701, Korea; E-Mail: lic9418@yd.ac.kr (I.C.L.); 4 Department of Herbal Medicinal Pharmacology, Daegu Haany University, Gyeongsan 712-715, Korea; E-Mails: lovemiracle5@naver.com (S.R.A.); 8519477@hanmail.net (S.J.J.)

**Keywords:** *Eremochloa ophiuroides*, *C*-glycosidic flavones, obesity, pancreatic lipase

## Abstract

Activity-guided fractionation of a methanolic extract of the leaves of *Eremochloa ophiuroides* (centipede grass) using a pancreatic lipase inhibitory assay led to the isolation and identification of a new *C*-glycosidic flavone, luteolin 6-*C*-β-D-boivinopyranoside (**1**), as well as eight known compounds. The structures of these compounds were established on the basis of interpretation of their spectroscopic data. Among these isolates, the *C*-glycosidic flavones **1**–**5** showed potent inhibitory effects on pancreatic lipase, with IC_50_ values ranging from 18.5 ± 2.6 to 50.5 ± 3.9 μM.

## 1. Introduction

Plants belonging to the genus *Eremochloa* (Poaceae) consist of eight species, mainly distributed throughout China, Southeast Asia, and South America. One of these, centipede grass (*Eremochloa ophiuroides*), is native to China and Southeast Asia and has become one of the most popular lawn grasses in South America [1]. Several *C*-glycosidic flavones and phenolic constituents have been isolated from this plant [2]. However, information on the bioactivity of this biomass is very limited.

Obesity is caused by an imbalance between energy intake and expenditure and is widely recognized as a major public health problem. Obesity can lead to a variety serious diseases, including hypertension, hyperlipidemia, arteriosclerosis, and type II diabetes [3]. Pancreatic lipase plays a key role for triglyceride absorption in the small intestine. This enzyme is secreted from the pancreas and hydrolyzes triglycerides into glycerol and fatty acids [4]. Therefore, pancreatic lipase inhibitors are considered to be a valuable therapeutic reagent for treating diet-induced obesity in humans. The success of orlistat [5], which is a specific pancreatic lipase inhibitor, has prompted research to identify new pancreatic lipase inhibitors derived from natural sources, such as the constituents of *Platycodin grandiflorum* [6], *Salvia officinalis* [7], *Salacia reticulate* [8], and oolong tea [9].

As part of our continuing search for bioactive natural products, the ethyl acetate (EtOAc)-soluble partition of a methanolic extract from the leaves of *E. ophiuroides* was found to exhibit an inhibitory effect against porcine pancreatic lipase. In the present work, activity-guided fractionation of the EtOAc-soluble portion using a pancreatic lipase inhibition assay led to the isolation of a new *C*-glycosidic flavone (**1**), along with several known compounds. Herein, we describe the isolation and structure elucidation of compound **1** and a biological evaluation of all metabolites obtained in this investigation.

## 2. Results and Discussion

Successive column chromatographic purification of the EtOAc-soluble fraction of the methanolic extract of *E. ophiuroides* led to the isolation and characterization of a new *C*-glycosylated flavone, luteolin 6-*C*-β-D-boivinopyranoside (**1**), along with eight known constituents. The known compounds were identified as orientin (**2**) [12], isoorientin (**3**) [12], derhamnosylmaysin (**4**) [13], isoorientin 2-*O*-α-L-rhamnoside (**5**) [14,15], luteolin (**6**) [16], chlorogenic acid (**7**) [17], methyl chlorogenate (**8**) [18,19], and caffeic acid (**9**) [20] from comparisons of their physicochemical properties and spectroscopic data (^1^H-, ^13^C-NMR, 2D NMR, and MS) with those of authentic samples and reference data. Compounds **3** and **4** were found to be the major metabolites of the EtOAc-soluble portion of the leaves of *E. ophiuroides*.

Compound **1** was obtained as a yellow amorphous powder, and FAB-MS gave a [M + H]^+^ ion peak at *m/z* 417. Its molecular formula, C_21_H_21_O_9_, was deduced from the molecular ion peak at *m/z* 417.1188 [M + H]^+^ in the HRFAB-MS, and from NMR data. The absorption maxima at 270 and 350 nm in the UV spectrum were attributed to a flavone nucleus [21]. The ^1^H-NMR spectrum of **1** (CD_3_OD, 40 °C, Table 1) exhibited broadened signals of 1,3,4-trisubtituted aromatic protons in the B ring at *δ*_H_ 7.44 (1H, d, *J* = 1.8 Hz, H-2’), 7.42 (1H, dd, *J* = 7.8, 1.8 Hz, H-6’), and 6.94 (1H, d, *J* = 7.8 Hz, H-5’), an isolated olefinic proton in the C ring at *δ*_H_ 6.60 (1H, br s, H-3), and an aromatic proton in the A ring at *δ*_H_ 6.52 (1H, br s, H-8). These signals suggest the presence of a luteolin (5,7,3’,4’-tetrahydroxy flavone) moiety in **1** [22], which was substantiated by the HMQC experiment. In addition to the aglycone signals, a characteristic anomeric signal at *δ*_H_ 5.53 (1H, dd, *J* = 12.6, 2.4 Hz, H-1”), three oxygen-bearing protons at *δ*_H_ 4.18 (1H, q, *J* = 6.6 Hz, H-5”) , 4.04 (1H, d-like, *J* = 3.0 Hz, H-3”), and 3.43 (1H, d-like, *J* = 3.0 Hz, H-4”), and a doublet methyl signal at *δ*_H_ 1.33 (3H, d, *J* = 6.6 Hz, H-6”) were observed, along with two aliphatic *gem*-protons at *δ*_H_ 2.31 (1H, ddd, *J* = 14.4, 12.6, 2.4 Hz, H-2”) and 1.77 (1H, dd, *J* = 14.4, 3.0 Hz, H-2”), indicating the presence of a dideoxy sugar moiety [23,24]. The presence of the dideoxy hexopyranose sugar unit was further suggested by ^13^C-NMR resonances at *δ*_C_ 32.8 (C-2”) and 17.4 (C-6”), which was supported by cross peaks (H-2”/H-1”, 3”, H-4”/H-3”, 5” and H-5”/6”) in the ^1^H-^1^H COSY, and long-range correlation of H-2”/C-1”, 3” and H-6”/C-5”, 4” in the HMBC spectrum. The location of the sugar moiety was elucidated by key HMBC correlations of H-1”/C-5, 6, 7 and clear NOESY cross peaks of H-8/H-2’, 6’ [23]. The chemical shifts of the C-6(*δ*_C_ 111.6) and C-8 (*δ*_C_ 96.0) carbons at the aglycone moiety indicated that the anomeric carbon of the dideoxy sugar was connected to C-6 through a *C*-linkage. The glycosidic linkage of the sugar moiety was determined to be β for deoxy hexose from the coupling constants for anomeric proton [25]. The ^1^H- and ^13^C-NMR spectra of **1** were similar to those of the known compound alternanthin [26], except for the absence of signals for a methoxy group at the C-3’ position. The relative stereochemistry of the sugar moiety was established on the basis of the magnitude of the coupling constants of the observed protons. Thus, the coupling constant between H-1” and H-2”_ax_ (12.6 Hz) indicated a *trans*-diaxial configuration, and the values between H-2”_eq_ and H-3” (3.0 Hz) and H-3” and H-4” (3.0 Hz) indicated an equatorial configuration. This arrangement was verified by the NOESY correlations of H-1”/H-5”, H-2”_eq_/H-3”, H-3”/H-4”, and H-4”/H-6”. According to the above spectroscopic data, the sugar moiety of **1** is boivinose (2,6-dideoxy-*xylo*-hexose) which was previously isolated from natural sources in both D- and L-enantiomers [27]. The absolute configuration of the sugar moiety was deduced to be D by comparing its specific optical rotation with that of alternanthin ([α]_D_ +86.2°) [26,28], with reference to semisynthetic β-methyl-D- ([α]_D_ –125°) and β-methyl-L-boivinopyranose ([α]_D_ +175°) [29]. Alternanthin B ([α]_D_ +86.9°) and luteolin 6-*C*-β-boivinopyranoside ([α]_D_ +25°) were isolated previously from *Alternanthera philoxeroides* [30] and *Pogonatherum crinitum* [31], but its absolute stereochemistry has not been elucidated thus far. Thus, the structure of the new compound **1** was fully elucidated as luteolin 6-*C*-β-D-boivinopyranoside. 

Pancreatic lipase plays an important role in lipid metabolism and is one of the most widely studied models for the evaluation of the potential efficacy of natural products as antiobesity agents [32]. The isolation and characterization of constituents **1**–**9** from *E. ophiuroides* was guided by their pancreatic lipase inhibitory activity, using orlistat as a positive control. The flavonoids **1**–**5** isolated in the highest quantities all had a *C*-glycosyl group in the A-ring and potently inhibited pancreatic lipase with IC_50_ values ranging from 18.5 ± 2.6 to 50.5 ± 3.9 μM. The known compound methyl chlorogenate (**8**) was found to be active (IC_50_ = 33.6 ± 2.0 μM), but the structurally related compounds chlorogenic acid (**7**) and caffeic acid (**9**) were inactive (IC_50_ > 200 μM). The inhibitory activity of luteolin (**6**), which does not have a *C*-glycosyl group on the A-ring of its flavonoid moiety, against pancreatic lipase was much weaker than those of *C*-glycosylated derivatives on their A-ring. Furthermore, the *C*-glycosylated flavone **2** at *C*-8 was more potent than a *C*-6 glycosylated flavone **3**. The presence of at least one *C*-glycosylated sugar moiety at the 6 or 8-position of the A-ring is essential for the enzyme inhibitory activity of the luteolin skeleton. The *C*-glycosylated sugar number and attached position in the A-ring appear to be particularly important for strong inhibitory activity. 

**Table 1:** 1D and 2D NMR spectroscopic data for compound **1** in CD_3_OD. ^a^

Position	*δ*_H_ (*J* in Hz)^b^	*δ*_C_ (mult.)		HMBC	NOESY
1					
2		166.5	C		
3	6.60 (1H, br s)	103.8	CH	2, 4, 10, 1’	2’
4		184.0	C=O		
5		158.7	C		
6		111.6	C		
7		164.6	C		
8	6.52 (1H, br s)	96.0	CH	6, 7, 8, 9	2’, 6’
9		158.3	C		
10		104.9	C		
1’		123.6	C		
2’	7.44 (1H, d, 1.8 Hz)	120.4	CH	2, 3, 1’	3
3’		147.1	C		
4’		151.1	C		
5’	6.94 (1H, d, 7.8 Hz)	116.8	CH	1’, 4’, 6’	6’
6’	7.42 (1H, dd, 7,8, 1.8 Hz)	114.2	CH	2, 1’, 5’	5’
1”	5.53 (1H, dd, 12.6, 2.4 Hz)	70.0	CH	5, 6, 7, 2”	5”
2_ax_”	2.31 (1H, ddd, 14.4, 12.6, 2.4 Hz)	32.8	CH_2_	1”, 3”	3”
2_eq_”	1.77 (1H, dd, 14.4, 3.0 Hz)			1”, 3”	3”
3”	4.04 (1H, d-like, 3.0 Hz)	68.8	CH	2”, 4”	2_ax_”, 2_eq_”, 4”
4”	3.43 (1H, d-like, 3.0 Hz)	70.6	CH	3”, 5”	3”
5”	4.18 (1H, q, 6.6 Hz)	72.7	CH	4”, 6”	1”, 6”
6”	1.33 (3H, d, 6.6 Hz)	17.4	CH_3_	4”, 5”	5”

^a^ ^1^H-NMR measured at 600 MHz, ^13^C-NMR measured at 150 MHz, TMS was used as the internal standard; ^b^ Chemical shifts are shown in the *δ* scale with *J* values (Hz) in parentheses.

**Table 2:** Pancreatic lipase inhibitory activity of compounds **1**–**9**.

Compound	IC_50_^a^ (μM)	Compound	IC_50_^a^ (μM)
**1**	50.5 ± 3.9	**6**	>200
**2**	31.6 ± 2.7	**7**	>200
**3**	44.6 ± 1.3	**8**	33.6 ± 2.0
**4**	25.9 ± 3.7	**9**	>200
**5**	18.5 ± 2.6	Orlistat ^b^	0.3 ± 0.2

^a^ IC_50_ values of compounds represent the concentration that caused a 50% reduction in enzyme activity. Inhibitory effects are expressed as the mean ± S.D. of quadruplicate experiments; ^b^ Orlistat was used as a positive control.

**Figure 1:**
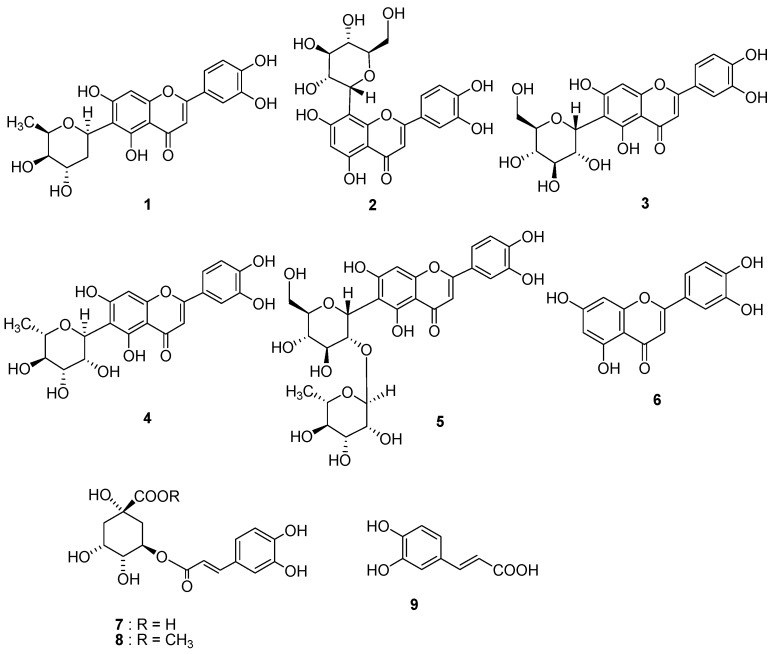
Structures of compounds **1**–**9** isolated from *E. ophiuroides*.

**Figure 2:**
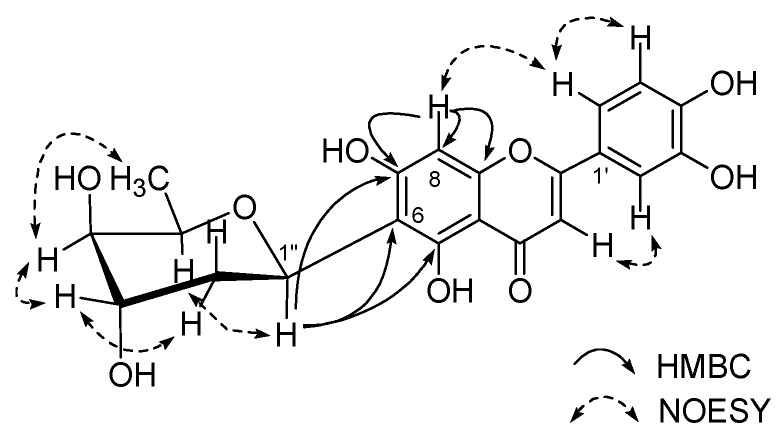
Selected HMBC and NOESY correlations of compound **1**.

## 3. Experimental

### 3.1. General

Optical rotations were measured with a JASCO DIP-4 digital polarimeter. UV spectra were acquired on a JASCO V-530 UV-vis spectrometer. The ^1^H- and ^13^C-NMR spectra were measured on a Varian VNS600 instrument operating at 600 and 150 MHz, respectively. The chemical shifts are given in *δ* (ppm) values relative to that of the solvent CD_3_OD (*δ*_H_ 3.35; *δ*_C_ 49.0) on a tetramethylsilane (TMS) scale. The standard pulse sequences programmed into the instruments were used for each 2D measurement. The *J*_CH_ value was set at 8 Hz in the HMBC spectra. FAB-MS using 3-nitrobenzyl alcohol as the matrix agent, including HRFAB-MS, was performed on a Micro Mass Auto SpecOA-TOF spectrometer. HPLC analysis was carried out on a YMC-Pack ODS A-302 column (4.6 mm i.d. × 150 mm; YMC Co., Ltd.) and the solvent system consisted of a linear gradient that started with 5 % (v/v) MeCN in 0.1% HCOOH/H_2_O (detection: UV 280 nm; flow rate: 1.0 mL/min; at 40 ^o^C), increased to 95 % MeCN over 30 min, and then increased to 100% MeCN over 5 min. At the end of the run, 100 % MeCN was allowed to flush the column for 5 min, and an additional 10 min of post-run time was set to allow for equilibration of the column. Column chromatography was carried out on Toyopearl HW-40 (coarse grade; Tosoh Co.), YMC GEL ODS AQ 120-50S (YMC Co., Ltd), and Sephadex LH-20 (Pharmacia Fine Chemicals Co., Ltd). Thin-layer chromatography (TLC) was performed on Kieselgel 60 F254 plates (0.2 mm layer thickness, Merck), and the spots were detected by ultraviolet irradiation (254, 365 nm) and by spraying with 10% H_2_SO_4_ reagent. 

### 3.2. Plant Material

In September, 2007, the leaves of *Eremochloa ophiuroides* were collected at the Advanced Radiation Technology Institute (ARTI), Jeongeup, Korea, and identified by Dr. Seung Sik Lee. A voucher specimen (KAJ-0053) was deposited at the Natural Product Chemistry Laboratory, College of Herbal Bio-industry, Daegu Hanny University.

### 3.3. Extraction and Isolation

Fresh milled *E. ophiuroides* plant material (5.0 kg) was extracted with MeOH (18 L × 3) at room temperature, and the solvent was evaporated *in vacuo*. The combined crude MeOH extract (102.1 g) was suspended in 20% MeOH (6 L), and then partitioned in turn with *n*-hexane (6 L × 3) and EtOAc (6 L × 3) to yield dried *n*-hexane- (15.3 g), EtOAc- (26.8 g), and H2O-soluble (64.1 g) residues. The EtOAc-soluble extract was found to be active in a pancreatic lipase inhibition assay with an IC_50_ value of 28.1 *μ*g/mL. Both the *n*-hexane-soluble and aqueous-soluble extracts were inactive in the enzyme-based bioassay system (IC_50_ > 200 *μ*g/mL). A portion (20.0 g) of the EtOAc extract was chromatographed on a Toyopearl HW-40 column (coarse grade; 3.2 cm i.d. × 50 cm) with H_2_O containing increasing amounts of MeOH in a stepwise gradient mode. The 20% MeOH eluate was subjected to column chromatography over a YMC GEL ODS AQ 120-50S column (1.1 cm i.d. × 32 cm) with aqueous MeOH, to yield pure compound **7** (43.1 mg; *t*_R_ = 13.9 min). The 50% MeOH eluate was subjected to a combination of chromatography over Sephadex LH-20 (1.1 cm i.d. × 43 cm) (with EtOH), and YMC GEL ODS AQ 120-50S (1.1 cm i.d. × 47 cm) (with aqueous MeOH) to yield pure compounds **4** (66.7 mg; *t*_R_ = 14.6 min), **5** (10.6 mg; *t*_R_ = 15.5 min), and **7** (37.3 mg). In similar fashion, the 70 % MeOH eluate was chromatographed over Sephadex LH-20 (1.1 cm i.d. × 43 cm) and YMC GEL ODS AQ 120-50S (1.1 cm i.d. × 45 cm), followed by preparative HPLC (YMC-Pack ODS A-302, 4.6 mm i.d.× 150 mm) using 20% aqueous MeCN to yield pure compounds **1** (25.0 mg; *t*_R_ = 17.8 min), **2** (15.9 mg; *t*_R_ = 16.2 min), **3** (35.1 mg; *t*_R_ = 14.8 min), **8** (10.4 mg; *t*_R_ = 15.2 min), and **9** (1.3 mg; *t*_R_ = 14.3 min). The MeOH eluate from the Toyopearl HW-40 (3.2 cm i.d. × 50 cm) was further fractionated by column chromatography on YMC GEL ODS AQ 120-50S (1.1 cm i.d. × 45 cm) with aqueous MeOH to yield pure compound **6** (2.1 mg; *t*_R_ = 17.7min).

Luteolin 6-*C*-β-D-boivinopyranoside (**1**): Yellow amorphous powder; [α]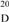 −35.0° (*c*, 1.0, MeOH); UV λ max MeOH nm (log *ε*): 212 (5.38), 222 (sh), 270 (2.55), 350 (3.03); ^1^H- and ^13^C-NMR, see Table 1; FAB-MS *m/z* 417 [M + H]^+^, HRFAB-MS *m/z* 417.1188 [M + H]^+^ (calcd for C_21_H_21_O_9_, 417.1186).

### 3.4. Assay for Pancreatic Lipase Activity

The ability of the compounds to inhibit porcine pancreatic lipase was evaluated using previously reported methods with a minor modification [10,11]. Briefly, an enzyme buffer was prepared by the addition of 30 μL (10 units) of a solution of porcine pancreatic lipase (Sigma, St. Louis, MO) in10 mM MOPS (morpholinepropanesulphonic acid), and 1 mM EDTA, pH 6.8 to 850 μL of Tris buffer (100 mM Tris-HC1 and 5 mM CaCl_2_, pH 7.0). Then, 100 μL of compound at the test concentration or Orlistat (Roche, Switzerland) was mixed with 880 μL of enzyme buffer, and incubated for 15 min at 37 ^o^C, then, 20 μL of the substrate solution [10 mM *p*-NPB (*p*-nitrophenylbutyrate) in dimethyl formamide] was added and the enzymatic reaction was allowed to proceed for 15 min at 37 ^o^C. Pancreatic lipase activity was determined by measuring the hydrolysis of *p*-NPB to *p*-nitrophenol at 405 nm using an ELISA reader (Tecan, Infinite F200, Austria). Inhibition of lipase activity was expressed as the percentage decrease in the OD when porcine pancreatic lipase was incubated with the test compounds.

## 4. Conclusions

In summary, we have investigated the pancreatic lipase inhibitory activity of a new flavonoid (**1**) and eight known compounds (**2**–**9**) isolated from the leaves of *E. ophiuroides*. Among the isolates, the *C*-glycosyl flavonoids were compared to estimate the optimal position and number of glycosyl groups on the flavone skeleton for significant and specific pancreatic lipase inhibitory activity. Compound **5**, a *C*-glycosyl flavone with two sugar moieties at C-6 of the A-ring, showed especially potent activity (IC_50_ = 18.5 ± 2.6 μM) when compared to the other tested compounds. 
